# *catena*-Poly[[bis­[1-(2-hydroxy­ethyl)-1*H*-tetra­zole-κ*N* ^4^]copper(II)]-di-μ-chlorido]: a powder study

**DOI:** 10.1107/S1600536808022137

**Published:** 2008-07-19

**Authors:** Ludmila S. Ivashkevich, Alexander S. Lyakhov, Tatiyana V. Serebryanskaya, Pavel N. Gaponik

**Affiliations:** a Research Institute for Physico-Chemical Problems, Belarusian State University, Leningradskaya Str. 14, Minsk 220030, Belarus

## Abstract

The crystal structure of the title polymeric complex, [CuCl_2_(C_3_H_6_N_4_O)_2_]_*n*_, was obtained by the Rietveld refinement from laboratory X-ray powder diffraction data collected at room temperature. The unique Cu^II^ ion lies on an inversion center and is in a slightly distorted octa­hedral coordination environment. In the hydroxy­ethyl group, all H atoms, the O atom and its attached C atom are disordered over two positions; the site occupancy factors are *ca* 0.6 and 0.4. The OH group is involved in an intra­molecular O—H⋯N hydrogen bond.

## Related literature

For related literature, see: Ivashkevich *et al.* (2001 ▶); Ivashkevich, Lyakhov *et al.* (2005 ▶); Ivashkevich, Voitekhovich & Lyakhov (2005 ▶); Stassen *et al.* (2002 ▶); Werner *et al.* (1985 ▶); Allen (2002 ▶); Virovets *et al.* (1995 ▶, 1996 ▶).
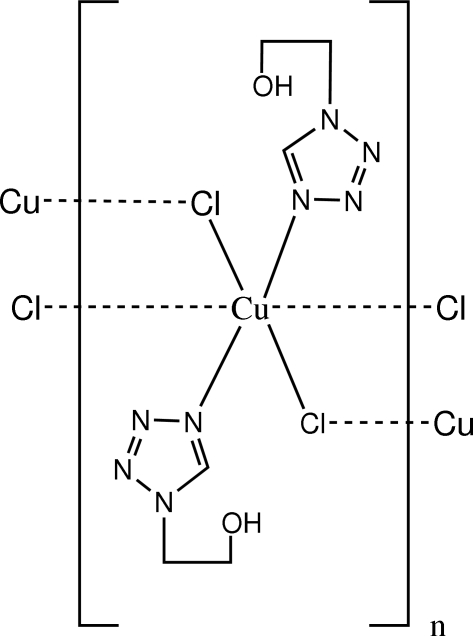


## Experimental

### 

#### Crystal data

[CuCl_2_(C_3_H_6_N_4_O)_2_]
*M* *_r_* = 362.69
Monoclinic, 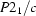

*a* = 13.3349 (11) Å
*b* = 6.7406 (6) Å
*c* = 7.3419 (5) Å
β = 105.450 (8)°
*V* = 636.08 (9) Å^3^
*Z* = 2
Cu *K*α radiation
*T* = 295 (2) K
Specimen shape: flat sheet
30 × 30 × 1 mm
Specimen prepared at 100 kPa
Specimen prepared at 295(2) K
Particle morphology: plate, green

#### Data collection

HZG-4A (Carl Zeiss, Jena) diffractometer
Specimen mounting: packed powder pellet
Specimen mounted in reflection mode
Scan method: step
2θ_min_ = 5.0, 2θ_max_ = 100.0°
Increment in 2θ = 0.02°

#### Refinement

*R*_p_ = 0.042
*R*_wp_ = 0.067
*R*_exp_ = 0.086
*R*_B_ = 0.029
*S* = 0.78
Wavelength of incident radiation: 1.5418 Å
Excluded region(s): none
Profile function: pseudo-Voigt
785 reflections
48 parameters
21 restraints
H-atom parameters constrained
Preferred orientation correction: Marsh–Dollase function (Marsh, 1932 ▶; Dollase, 1986 ▶)

### 

Data collection: local program; cell refinement: *FULLPROF* (Rodríguez-Carvajal, 2001 ▶); data reduction: local program; program(s) used to refine structure: *FULLPROF* and *SHELXL97* (Sheldrick, 2008 ▶); molecular graphics: *ORTEP-3 for Windows* (Farrugia, 1997 ▶) and *PLATON* (Spek, 2003 ▶); software used to prepare material for publication: *FULLPROF* and *PLATON*.

## Supplementary Material

Crystal structure: contains datablocks global, I. DOI: 10.1107/S1600536808022137/lh2648sup1.cif

Rietveld powder data: contains datablocks I. DOI: 10.1107/S1600536808022137/lh2648Isup2.rtv

Additional supplementary materials:  crystallographic information; 3D view; checkCIF report

## Figures and Tables

**Table:** 

Cu—Cl	2.234 (7)
Cu—N4	1.979 (10)
Cu—Cl^i^	3.008 (7)
Cu—Cu^ii^	4.9835 (4)

**Table:** 

Cl—Cu—N4	89.8 (7)
Cl—Cu—Cl^i^	90.8 (2)
Cl—Cu—Cl^iii^	180
Cl—Cu—N4^iii^	90.2 (7)
Cl—Cu—Cl^iv^	89.2 (2)
Cl^i^—Cu—N4	92.6 (5)
N4—Cu—N4^iii^	180
Cl^iv^—Cu—N4	87.4 (5)
Cu—Cl—Cu^ii^	143.5 (2)

Symmetry codes: (i) 
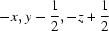
; (ii) 
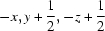
; (iii) 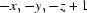; (iv) 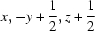.

**Table 2:** Hydrogen-bond geometry (Å, °)

*D*—H⋯*A*	*D*—H	H⋯*A*	*D*⋯*A*	*D*—H⋯*A*
O1—H1⋯N2	0.82	2.35	3.08 (3)	149
O2—H2⋯N2	0.82	2.46	3.02 (3)	126
C5—H5⋯Cl^ii^	0.93	2.72	3.34 (2)	126

Symmetry code: (ii) 
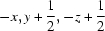
.

**Fig. 1.**
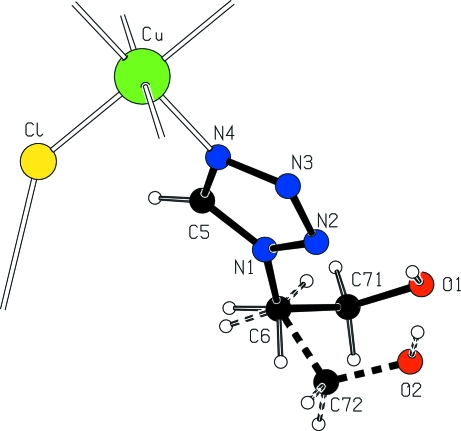
The asymmetric unit of (I) with the atomic numbering scheme. 2-hydroxyethyl substituent is shown as disordered over two positions.

**Fig. 2.**
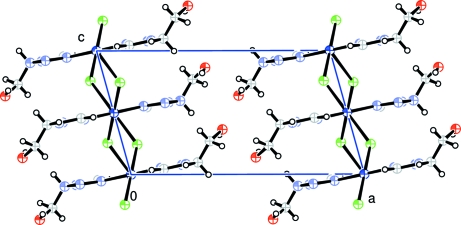
Layered structure of (I), viewed along the b axis. Disordered 2-hydroxyethyl substituent is shown only in position with occupancy factor of 0.562 (12).

**Fig. 3.**
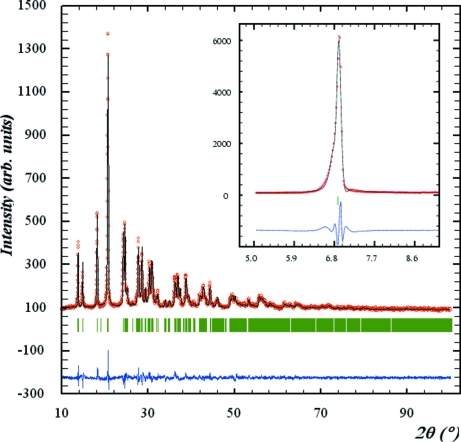
The Rietveld plot, showing the observed (circles), calculated (line) and difference patterns for (I). The reflection positions are shown above the difference pattern.

## supplementary crystallographic information

### Comment

Complexes of copper(II) chloride with substituted tetrazoles attract attention because of their magnetic bahaviour at low temperatures. Among them, there are layered coordination polymers with square grids of only Cu and Cl atoms, of the composition CuCl_2_*L*_2_, where *L* = 1-ethyltetrazole (Virovets *et al.*, 1995), 1-allyltetrazole (Virovets *et al.*, 1996), 1-(2-azidoethyl)terazole (Ivashkevich *et al.*, 2001), 1-(2-chloroethyl)tetrazole (Stassen *et al.*, 2002), 1-benzyltetrazole (Ivashkevich, Voitekhovich & Lyakhov, 2005), and 1-methyltetrazole (Ivashkevich, Lyakhov, Ivashkevich, Degtyarik & Gaponik, 2005). These compounds crystallize in the space group *P*2_1_/*c* and are isotypic. Here, we present another example, poly[[bis(1-(2-hydroxyethyl)tetrazole-*N*^4^)copper(II)]-di-µ-chloro], (I), (Fig. 1). As it is difficult to obtain single crystals for structural analysis, the compound (I) was investigated by X-ray powder diffraction.

The Cu atom lies on inversion center and shows a slightly distorted octahedral coordination environment. Equatorial sites are occupied by two *trans* positioned N atoms and two Cl atoms; Cl atoms lying in axial positions are essentially more distant from the Cu atom (Table 1). Each Cl atom is a bridge between the neighbouring Cu atoms, forming two different in length Cu—Cl bonds, with Cu—Cl—Cu angle of 143.4 (2)°. These bonds are responsible for the formation of polymeric layers parallel to the yz plane (Fig. 2). Within a layer, the shortest Cu···Cu distance is 4.9835 (4) Å, whereas between two neighbouring layers, the closest Cu centers are separated by cell dimension a. Only van der Waals interactions are between the layers.

The 2-hydroxyethyl substituent at the tetrazole ring atom N1 was found to be disordered over two positions, with almost equal occupancies of 0.562 (12) for C71—O1 and 0.438 (12) for C72—O2 (Fig. 1). For both positions, OH groups are involved in intramolecular hydrogen bonds O—H···N. There are also hydrogen bonds C—H···Cl (Table 2).

### Experimental

A solution, containing 2.13 g (0.0125 mol) of CuCl_2_.2H_2_O in 75 ml of ethanol, was added to a slightly heated solution of 1-(2-hydroxyethyl)tetrazole (2.85 g, 0.025 mol) in a solvent mixture (45 ml of ethanol and 30 ml of n-buthanol), with stirring at room temperature. After stirring the reaction mixture for 10 min, the obtained green crystals of (I) were filtered off, air dried and recrystallized from (ethanol—n-buthanol) mixture (*v*/*v* = 4:1) [3.55 g, yield 78.3%]. Calc.(%): Cu 17.52, Cl 19.59. Found (%): Cu 18.2, Cl 20.1.

### Refinement

The monoclinic unit-cell dimensions of (I) were determined with the indexing program TREOR90 (Werner *et al.*, 1985). The obtained values indicated isotypism of (I) with layered coordination polymers CuCl_2_*L*_2_ (*L* = 1-alkyltetrazole) that crystallize in the monoclinic space group *P*2_1_/*c*. This space group and the atomic coordinates of CuCl_2_*L*_2_ with *L* = 1-ethyltetrazole (Virovets *et al.*, 1995) were used as starting parameters for the Rietveld refinement with the FULLPROF program (Rodríguez-Carvajal, 2001). Background intensity was found by Fourier filtering technique as implemented in the FULLPROF program, under visual inspection of the resulting background curve. Correction for profile asymmetry was made for reflections up to 2θ=30°. A Marsh-Dollase correction of intensities for [100] preferred orientation of plate-like grains in the sample (Marsh, 1932; Dollase, 1986) was applied.

The Rietveld refinement, performed primarily by using individual isotropic displacement parameters for non-H atoms, revealed rather high values of B_iso_ for atoms of C—O fragment. From this fact an assumption was made that C—O fragment was disordered over two positions. It was confirmed in subsequent refinement by introducing disorder positions for the above C and O atoms. In final refinement, all non-H atoms were refined with overall B_iso_ parameter.

All H atoms were placed in geometrically calculated positions, using the program *SHELXL97* (Sheldrick, 2008), with displacement parameter B_iso_(H)=1.2B_iso_(C) for H atom at C5 tetrazole ring atom and B_iso_(H)=1.5B_iso_(C,*O*) for the methylene and hydroxyl groups.

Soft restraints on some interatomic distances and bond angles of ligand molecule, based on a geometric analysis of a large number of 1-substituted tetrazoles (Cambridge Structural Database, version 5.29 of November 2007; Allen, 2002), were used in the Rietved refinement. Observed, calculated and difference difraction patterns are shown in Fig. 3.

### Crystal data

**Table:**

[CuCl_2_(C_3_H_6_N_4_O)_2_]	*F*_000_ = 366.0
*M**_r_* = 362.69	*D*_x_ = 1.894 Mg m^−^^3^
Monoclinic, *P*2_1_/*c*	Cu *K*α radiation λ = 1.5418 Å
Hall symbol: -P 2ybc	*T* = 295 (2) K
*a* = 13.3349 (11) Å	Specimen shape: flat sheet
*b* = 6.7406 (6) Å	30 × 30 × 1 mm
*c* = 7.3419 (5) Å	Specimen prepared at 100 kPa
β = 105.450 (8)º	Specimen prepared at 295(2) K
*V* = 636.08 (9) Å^3^	Particle morphology: plate, green
*Z* = 2	

### Data collection

**Table:**

HZG-4A (Carl Zeiss, Jena) diffractometer	*T* = 295 K
Monochromator: Ni filtered	2θ_min_ = 5.00, 2θ_max_ = 100.00º
Specimen mounting: packed powder pellet	Increment in 2θ = 0.02º
Specimen mounted in reflection mode	Increment in 2θ = 0.02º
Scan method: step	

### Refinement

**Table:**

Refinement on *I*_net_	Excluded region(s): none
Least-squares matrix: full with fixed elements per cycle	Profile function: psevdo-Voigt
*R*_p_ = 0.042	48 parameters
*R*_wp_ = 0.067	21 restraints
*R*_exp_ = 0.086	H-atom parameters constrained
*R*_B_ = 0.029	Weighting scheme based on measured s.u.'s ?
*S* = 0.78	(Δ/σ)_max_ = 0.02
Wavelength of incident radiation: 1.5418 Å	Preferred orientation correction: Marsh–Dollase function (Marsh, 1932; Dollase, 1986)

### Fractional atomic coordinates and isotropic or equivalent isotropic displacement parameters (Å^2^)

**Table:**

	*x*	*y*	*z*	*U*_iso_*/*U*_eq_	Occ. (<1)
Cu	0.00000	0.00000	0.50000	0.0309 (10)*	
Cl	−0.0624 (4)	0.2048 (11)	0.2569 (10)	0.0309 (10)*	
N1	0.24708 (16)	0.3706 (2)	0.583 (4)	0.0309 (10)*	
N2	0.2982 (2)	0.19676 (13)	0.595 (3)	0.0309 (10)*	
N3	0.2308 (2)	0.05581 (18)	0.577 (3)	0.0309 (10)*	
N4	0.1358 (4)	0.1377 (3)	0.553 (4)	0.0309 (10)*	
C5	0.14654 (16)	0.3314 (4)	0.545 (5)	0.0309 (10)*	
H5	0.09292	0.42403	0.51621	0.0309 (10)*	
C6	0.3006 (6)	0.5643 (10)	0.5973 (16)	0.0309 (10)*	
H61A	0.24602	0.66346	0.56434	0.0309 (10)*	0.562 (12)
H61B	0.33551	0.56618	0.49731	0.0309 (10)*	0.562 (12)
C71	0.3778 (18)	0.641 (2)	0.768 (3)	0.0309 (10)*	0.562 (12)
H71A	0.41791	0.74586	0.73283	0.0309 (10)*	0.562 (12)
H71B	0.34071	0.69633	0.85467	0.0309 (10)*	0.562 (12)
O1	0.4451 (15)	0.489 (3)	0.862 (5)	0.0309 (10)*	0.562 (12)
H1	0.42627	0.38207	0.81168	0.0309 (10)*	0.562 (12)
H62A	0.31564	0.60902	0.72756	0.0309 (10)*	0.438 (12)
H62B	0.25331	0.65967	0.52039	0.0309 (10)*	0.438 (12)
C72	0.3987 (12)	0.565 (7)	0.539 (4)	0.0309 (10)*	0.438 (12)
H72A	0.38957	0.48960	0.42321	0.0309 (10)*	0.438 (12)
H72B	0.41727	0.69967	0.51599	0.0309 (10)*	0.438 (12)
O2	0.479 (2)	0.480 (6)	0.684 (5)	0.0309 (10)*	0.438 (12)
H2	0.46854	0.35907	0.68929	0.0309 (10)*	0.438 (12)

### Geometric parameters (Å, °)

**Table:**

Cu—Cl	2.234 (7)	N3—N4	1.350 (11)
Cu—N4	1.979 (10)	N4—C5	1.316 (4)
Cu—Cl^i^	3.008 (7)	C6—C71	1.49 (2)
Cu—Cl^ii^	2.234 (7)	C6—C72	1.48 (2)
Cu—N4^ii^	1.979 (10)	C5—H5	0.9300
Cu—Cl^iii^	3.008 (7)	C6—H61B	0.9700
O1—C71	1.41 (3)	C6—H62A	0.9700
O2—C72	1.42 (5)	C6—H61A	0.9700
O1—H1	0.8200	C6—H62B	0.9700
O2—H2	0.8200	C71—H71A	0.9700
N1—N2	1.346 (6)	C71—H71B	0.9700
N1—C5	1.321 (15)	C72—H72A	0.9700
N1—C6	1.478 (8)	C72—H72B	0.9700
N2—N3	1.291 (7)	Cu—Cu^iv^	4.9835 (4)
			
Cl—Cu—N4	89.8 (7)	N1—C6—C72	115 (2)
Cl—Cu—Cl^i^	90.8 (2)	N1—C6—C71	125.5 (14)
Cl—Cu—Cl^ii^	180	O1—C71—C6	111.5 (15)
Cl—Cu—N4^ii^	90.2 (7)	O2—C72—C6	110 (2)
Cl—Cu—Cl^iii^	89.2 (2)	N1—C5—H5	126.00
Cl^i^—Cu—N4	92.6 (5)	N4—C5—H5	126.00
Cl^ii^—Cu—N4	90.2 (7)	N1—C6—H61B	106.00
N4—Cu—N4^ii^	180	N1—C6—H62A	108.00
Cl^iii^—Cu—N4	87.4 (5)	N1—C6—H62B	108.00
Cl^i^—Cu—Cl^ii^	89.2 (2)	C71—C6—H61A	106.00
Cl^i^—Cu—N4^ii^	87.4 (5)	C71—C6—H61B	106.00
Cl^i^—Cu—Cl^iii^	180.00	H61A—C6—H61B	106.00
Cl^ii^—Cu—N4^ii^	89.8 (7)	C72—C6—H62A	109.00
Cl^ii^—Cu—Cl^iii^	90.8 (2)	C72—C6—H62B	109.00
Cl^iii^—Cu—N4^ii^	92.6 (5)	H62A—C6—H62B	107.00
Cu—Cl—Cu^iv^	143.5 (2)	N1—C6—H61A	106.00
C71—O1—H1	109.00	O1—C71—H71A	109.00
C72—O2—H2	109.00	O1—C71—H71B	109.00
N2—N1—C6	122.6 (7)	C6—C71—H71B	109.00
C5—N1—C6	129.4 (6)	H71A—C71—H71B	108.00
N2—N1—C5	107.9 (5)	C6—C71—H71A	109.00
N1—N2—N3	107.9 (6)	C6—C72—H72A	110.00
N2—N3—N4	108.4 (3)	C6—C72—H72B	110.00
Cu—N4—N3	127.6 (3)	O2—C72—H72A	110.00
N3—N4—C5	107.5 (8)	O2—C72—H72B	109.00
Cu—N4—C5	124.1 (9)	H72A—C72—H72B	108.00
N1—C5—N4	107.8 (9)		

Symmetry codes: (i) −*x*, *y*−1/2, −*z*+1/2; (ii) −*x*, −*y*, −*z*+1; (iii) *x*, −*y*+1/2, *z*+1/2; (iv) −*x*, *y*+1/2, −*z*+1/2.

### Hydrogen-bond geometry (Å, °)

**Table:**

*D*—H···*A*	*D*—H	H···*A*	*D*···*A*	*D*—H···*A*
O1—H1···N2	0.8200	2.3500	3.08 (3)	149.00
O2—H2···N2	0.8200	2.4600	3.02 (3)	126.00
C5—H5···Cl^iv^	0.9300	2.7200	3.34 (2)	126.00

Symmetry codes: (iv) −*x*, *y*+1/2, −*z*+1/2.

## References

[bb1] Allen, F. H. (2002). *Acta Cryst.* B**58**, 380–388. 10.1107/s0108768102003890 12037359

[bb2] Dollase, W. A. (1986). *J. Appl. Cryst.* **19**, 267–272.

[bb3] Farrugia, L. J. (1997). *J. Appl. Cryst.* **30**, 565.

[bb4] Ivashkevich, D. O., Lyakhov, A. S., Gaponik, P. N., Bogatikov, A. N. & Govorova, A. A. (2001). *Acta Cryst.* E**57**, m335–m337.

[bb5] Ivashkevich, L. S., Lyakhov, A. S., Ivashkevich, D. O., Degtyarik, M. M. & Gaponik, P. N. (2005). *Russ. J. Inorg. Chem.* **50**, 78–82.

[bb6] Ivashkevich, D. O., Voitekhovich, S. V. & Lyakhov, A. S. (2005). XXII International Chugaev Conference on Coordination Chemistry, Kishinev, 2005. Book of Abstracts, p. 371.

[bb7] Marsh, A. (1932). *Z. Kristallogr.* **81**, 285–297.

[bb8] Rodríguez-Carvajal, J. (2001). *FULLPROF* CEA/Saclay, France.

[bb9] Sheldrick, G. M. (2008). *Acta Cryst.* A**64**, 112–122. 10.1107/S0108767307043930 18156677

[bb10] Spek, A. L. (2003). *J. Appl. Cryst.* **36**, 7–13.

[bb11] Stassen, A. F., Kooijman, H., Spek, A. L., Jos de Jongh, L., Haasnoot, J. G. & Reedijk, J. (2002). *Inorg. Chem.* **41**, 6468–6473. 10.1021/ic0257470 12444792

[bb12] Virovets, A. V., Baidina, I. A., Alekseev, V. I., Podberezskaya, N. V. & Lavrenova, L. G. (1996). *Zh. Strukt. Khim.* **37**, 330–336.

[bb13] Virovets, A. V., Podberezskaya, N. V., Lavrenova, L. G. & Bikzhanova, G. A. (1995). *Acta Cryst.* C**51**, 1084–1087.

[bb14] Werner, P.-E., Eriksson, L. & Westdahl, M. (1985). *J. Appl. Cryst.* **18**, 367–370.

